# Trends in circulatory-system mortality among adults with arthrosis in the U.S.

**DOI:** 10.1038/s41598-026-52258-4

**Published:** 2026-05-12

**Authors:** Yu Liu, Yuzhe He, Jiahui Weng, Changsong Chen, Lidong Wu

**Affiliations:** 1 Department of Orthopedic Surgery, The Second Affiliated Hospital, Zhejiang University School of Medicine, Hangzhou, Zhejiang China; 2 Department of Orthopedics, Hospital of Zhejiang People’s Armed Police (PAP), Hangzhou, Zhejiang China; 3 Orthopedics Research Institute of Zhejiang University, Hangzhou City, Zhejiang Province PR China; 4 Clinical Research Centre of Motor System Disease of Zhejiang Province, Hangzhou City, PR China; 5 Zhejiang Key Laboratory of Motor System Disease Precision Research and Therapy, Hangzhou City, Zhejiang Province PR China

**Keywords:** Arthrosis, Osteoarthritis, Circulatory system diseases, Cardiovascular mortality, Multiple cause of death, Mortality trends, Health disparities, Joinpoint regression, Diseases, Health care, Medical research, Risk factors

## Abstract

Arthrosis is prevalent in later life and commonly coexists with cardiovascular and other circulatory conditions. We quantified U.S. mortality in which diseases of the circulatory system (ICD-10: I00–I99) were the underlying cause of death, and arthrosis (M15–M19) was recorded as a contributing cause among adults aged ≥ 55 years. We used the CDC WONDER multiple-cause-of-death database (1999–2023). Age-adjusted mortality rates (AAMRs; standardised to the 2000 U.S. population) and age-specific crude mortality rates were calculated. Temporal trends were evaluated using joinpoint regression to estimate the average annual percent change (AAPC). Analyses were stratified by sex, age group, race/ethnicity, census region, urbanisation, and state. Deaths decreased from 7,290 in 1999 to 2,022 in 2023 (− 72.26%). The AAMR declined from 12.67 to 2.27 per 100,000, with an AAPC of − 6.87% (95% CI: −7.64 to − 6.09); Joinpoint regression revealed a non-linear decline with distinct temporal phases separated by identifiable inflection points. In 2023, the AAMR was higher in females than males (2.63 vs. 1.72 per 100,000) and was concentrated among adults aged ≥ 85 years (21.10 per 100,000). Nonmetropolitan areas had higher AAMRs than metropolitan areas; however, direct comparisons were restricted to 2020, the most recent year with available estimates for both categories. Hispanic (1.34) and non-Hispanic Other (1.02) groups had lower AAMRs than non-Hispanic Black (2.50) and non-Hispanic White (2.48) groups. State-level AAMRs in 2023 ranged from 1.17 (Arizona) to 5.83 (Oregon). U.S. circulatory-system mortality with coexisting arthrosis declined substantially from 1999 to 2023; however, marked demographic and geographic heterogeneity persisted. Continued surveillance is warranted and may inform cardiovascular risk assessment in older adults with arthrosis.

## Introduction

Diseases of the circulatory system remain a major source of mortality in the public health context, with the burden concentrated in later life; accordingly, we focused on adults aged ≥ 55 years. In recent national statistics, cardiovascular disease continues to be the leading cause of death, and the majority of heart disease deaths occur among older adults^1,2^. In parallel, arthrosis is highly prevalent in older populations and represents a substantial driver of disability and healthcare utilization^3^. The ICD-10 codes M15–M19 capture arthrosis, which predominantly reflects osteoarthritis in this age group; therefore, the term “osteoarthritis” is used when referring to the broader literature context. Because pain control is central to osteoarthritis management, NSAID use is mentioned here as a potential factor to motivate future research, but our study does not include individual-level medication data and does not imply a causal relationship with the observed mortality trends^4,5^. Evidence from large-scale meta-analyses and real-world individual-patient data suggests that the risk of myocardial infarction and other vascular outcomes differs across NSAID agents and may be detectable even with relatively short-term use^6,7^. Randomised evidence in arthritis populations at increased cardiovascular risk further highlights the clinical importance of balancing analgesic benefits with cardiovascular, gastrointestinal, and renal safety profiles^8^. Together, these considerations motivate closer epidemiologic surveillance at the intersection of circulatory-system mortality and arthrosis among older adults.

Many national mortality trend analyses emphasise single disease categories or rely primarily on the underlying cause of death, which may understate the role of coexisting chronic conditions recorded on death certificates^9^. Multiple cause-of-death data—available through the Centres for Disease Control and Prevention Wide-Ranging Online Data for Epidemiologic Research (CDC WONDER) system—capture both underlying and contributing causes and have been used to characterise cardiovascular mortality patterns in populations with complex comorbidity profiles^9,10^. Nevertheless, population-level evidence describing long-term temporal trends, demographic disparities, and geographic heterogeneity in circulatory-system deaths occurring in the presence of arthrosis remains limited. To address this gap, we used the U.S. Multiple Cause of Death database (1999–2023) to quantify mortality with diseases of the circulatory system (ICD-10 I00–I99) as the underlying cause and arthrosis (ICD-10 M15–M19) recorded as a coexisting condition among adults aged ≥ 55 years. We further assessed temporal patterns using age-adjusted and age-specific mortality metrics, and evaluated heterogeneity by sex, age group, race/ethnicity, and geographic context (state, census region, and urbanisation). By clarifying the epidemiology of circulatory-system mortality occurring alongside arthrosis in older adults, this work aims to inform integrated risk-reduction strategies and support safer analgesic decision-making in populations with high baseline cardiovascular risk. To our knowledge, this is one of the first national studies to use multiple-cause-of-death data to characterize circulatory-system mortality in the presence of arthrosis, providing a more comprehensive assessment of multimorbidity patterns than analyses based solely on underlying cause of death.

## Methods

### Study design and cohort

We conducted a serial cross-sectional, population-based mortality study using the Centres for Disease Control and Prevention (CDC) Wide-ranging Online Data for Epidemiologic Research (CDC WONDER) Multiple Cause of Death (MCD) public-use files from 1999 to 2023^9^. The manuscript was prepared in accordance with the Strengthening the Reporting of Observational Studies in Epidemiology (STROBE) guidance for observational research^11^.

We identified decedents aged ≥ 55 years whose underlying cause of death (UCOD) was classified as diseases of the circulatory system (ICD-10: I00–I99) and who had arthrosis recorded anywhere among the multiple causes of death (ICD-10: M15–M19). Age was categorised into 10-year intervals: 55–64, 65–74, 75–84, and ≥ 85 years.

### Data extraction

Using the CDC WONDER Multiple Cause of Death (MCD) interface, we extracted annual death counts and population denominators for decedents aged ≥ 55 years meeting the study definition (underlying cause of death ICD-10: I00–I99 and arthrosis recorded anywhere among multiple causes of death ICD-10: M15–M19). Outputs were generated for the overall cohort and for stratified analyses by sex, age group, and race/ethnicity as defined within CDC WONDER for each study year. Race/ethnicity data were harmonized across the study period to ensure comparability. CDC WONDER provides bridged-race estimates for 1999–2017 and single-race estimates from 2018 onward. All data were recategorized into a unified classification scheme (non-Hispanic White, non-Hispanic Black, Hispanic, and non-Hispanic Other) for analysis. Mortality trends for American Indian/Alaska Native and Asian/Pacific Islander groups were not assessed due to low death counts, which may yield unreliable estimates.

Geographic analyses were performed by state of residence and summarised by U.S. Census region (Northeast, Midwest, South, and West)^12^. County-level urbanisation was derived from the NCHS Urban–Rural Classification Scheme for Counties (2013 scheme), as provided in the CDC WONDER MCD files^13^. At the time of data extraction, CDC WONDER did not return an age-adjusted metropolitan estimate for 2023; where reported, the metropolitan AAMR shown for the most recent year corresponds to the most recent available estimate (2020), and this is explicitly noted in the Results and Table footnotes. The CDC WONDER Multiple Cause of Death data were accessed for research purposes in December 2025. The authors did not have access to information that could identify individual participants at any time.

### Statistical analysis

Mortality was quantified using age-adjusted mortality rates (AAMRs) per 100,000 population, calculated by direct standardisation to the 2000 U.S. standard population. For age-stratified analyses, we reported age-specific crude mortality rates for each age group (55–64, 65–74, 75–84, and ≥ 85 years). Percent changes in deaths and mortality rates from 1999 to 2023 were computed as: [ ( (value_{2023}− value_{1999})/value_{1999})×100].

Age-adjusted rates and their 95% CIs were obtained from CDC WONDER; APC/AAPC estimates and 95% CIs were derived from the joinpoint models.

Temporal trends in annual mortality rates were evaluated with joinpoint regression using log-transformed annual rates. We allowed up to four joinpoints, and the number of joinpoints was selected using permutation tests as described by Kim et al.^14^. For each time segment, annual percent change (APC) and 95% confidence intervals (CIs) were estimated. Overall trend summaries for prespecified intervals (including 1999–2023) were reported as average annual percent change (AAPC), calculated using the weighted approach proposed by Clegg et al.^15^. Statistical significance was defined as two-sided *P* < 0.05 when applicable.

### Ethics statement

This study used publicly available, de-identified data from the CDC WONDER Multiple Cause of Death database (https://wonder.cdc.gov/mcd.html). According to the Institutional Review Board of Zhejiang University School of Medicine, studies based on publicly available, de-identified data are exempt from institutional review board approval and informed consent requirements. Therefore, ethical approval and informed consent were not required for this study.

## Results

**Fig. 1:**
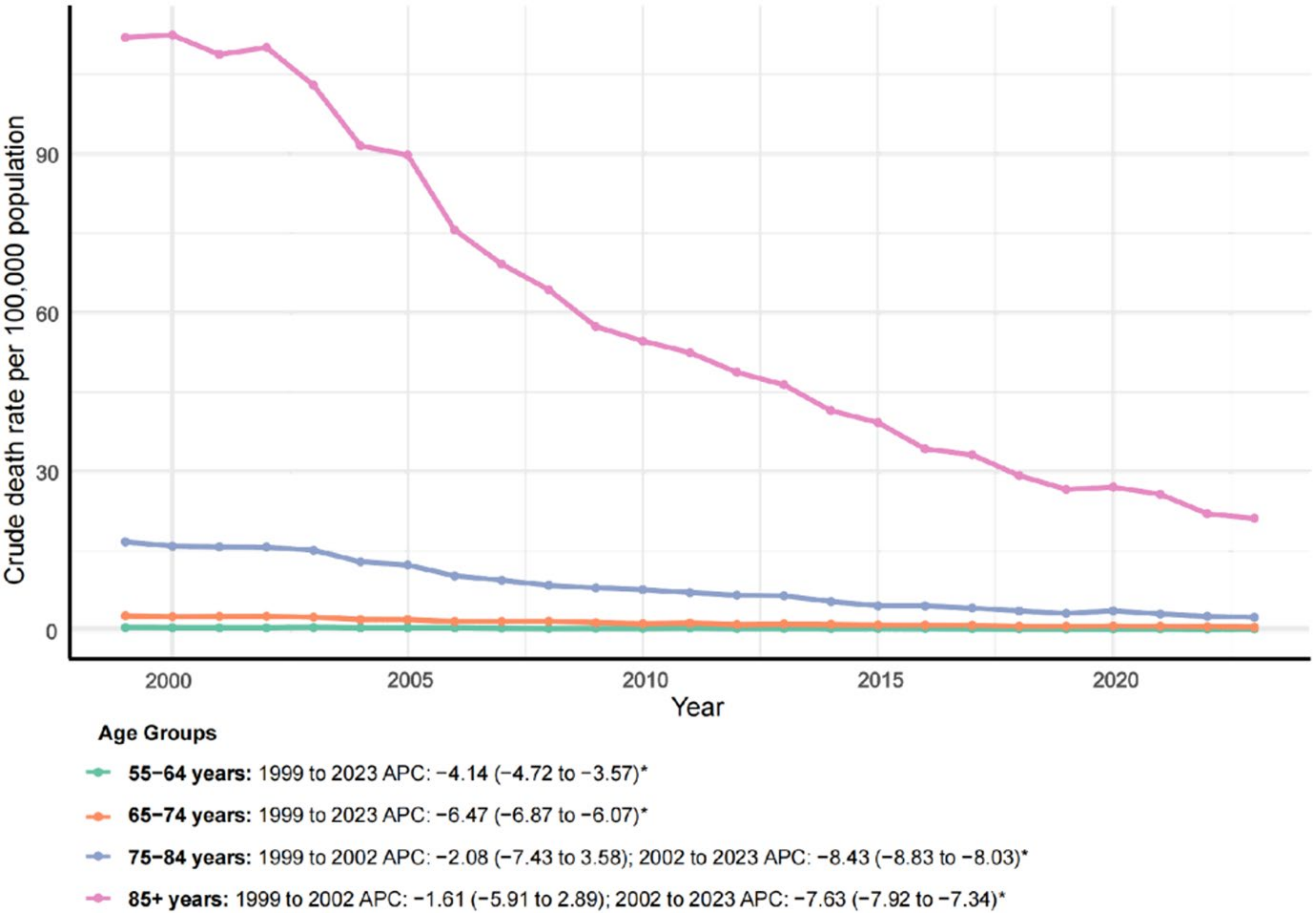
Age-specific mortality rates for circulatory diseases with arthrosis, 1999–2023. Age-specific (crude) mortality rates per 100,000 population were calculated for deaths with an underlying cause of death of diseases of the circulatory system (ICD-10: I00–I99) and co-occurring arthrosis (ICD-10: M15–M19) among U.S.adults aged ≥ 55 years during 1999–2023, stratified by age group. An asterisk (*) indicates that the annual percent change (APC) is significantly different from zero at α = 0.05.APC, annual percent change; CI, confidence interval.

Between 1999 and 2023, deaths involving coexisting diseases of the circulatory system (UICD: I00–I99) and arthrosis (M15–M19) declined from 7,290 to 2,022 (− 72.26%), and the AAMR decreased from 12.67 (95% CI: 12.38–12.96) to 2.27 (2.17–2.37) per 100,000 population (Table 1). The overall trend showed a decrease (AAPC − 6.87%, 95% CI: −7.64 to − 6.09; *P* < 0.001; Table 1). Joinpoint regression indicated that the decline in mortality was non-linear, with distinct inflection points observed over the study period (Fig. 1). In 2023, rates were higher in females than males (2.63 vs. 1.72 per 100,000; Table 1), were concentrated among adults aged ≥ 85 years (21.10 per 100,000; Table 1), and were higher in nonmetropolitan than metropolitan areas (Table 1).

**Table 1:** Deaths, age-adjusted mortality rates (AAMRs), and average annual percent change (AAPC) for deaths with underlying cause of death of diseases of the circulatory system (UCOD: I00–I99) and co-occurring arthrosis (ICD-10: M15–M19) among U.S. adults aged ≥ 55 years, 1999–2023, overall and by sex, census region, race/ethnicity, urbanization, and age group.

Characteristic	Deaths			AAMR		
	Deaths_1999	Deaths_2023	percent. change	AAMR_1999	AAMR_2023	AAPC (95% CI)
Both	7290	2022	-72.26	12.67 (12.38 to 12.96)	2.27 (2.17 to 2.37)	-6.87 (-7.64 to-6.09*)
Sex						
Female	5486	1384	-74.77	14.17 (13.80 to 14.55)	2.63 (2.49 to 2.77)	-6.86 (-7.62 to-6.09*)
Male	1804	638	-64.63	9.14 (8.71 to 9.57)	1.72 (1.59 to 1.86)	-6.53 (-7.13 to-5.92*)
Census Region						
Northeast	1354	428	-68.39	10.76 (10.19 to 11.33)	2.54 (2.30 to 2.78)	-5.92 (-7.30 to-4.52*)
Midwest	2471	467	-81.10	17.32 (16.63 to 18.00)	2.51 (2.28 to 2.73)	-7.21 (-7.66 to-6.76*)
South	2045	648	-68.31	10.35 (9.90 to 10.80)	1.93 (1.78 to 2.08)	-6.67 (-7.52 to-5.82*)
West	1420	479	-66.27	12.83 (12.16 to 13.50)	2.44 (2.22 to 2.66)	-6.66 (-7.39 to-5.93*)
Race/ethnicity						
Hispanic	180	104	-42.22	8.27 (7.05 to 9.50)	1.34 (1.08 to 1.60)	-7.48 (-8.06 to-6.89*)
NH Black	515	202	-60.78	11.80 (10.78 to 12.82)	2.50 (2.15 to 2.86)	-6.77 (-7.94 to-5.60*)
NH White	6498	1649	-74.62	13.01 (12.70 to 13.33)	2.48 (2.36 to 2.60)	-6.67 (-7.34 to-5.99*)
NH Other	78	62	-20.51	6.64 (5.23 to 8.33)	1.02 (0.78 to 1.31)	-6.68 (-8.09 to-5.25*)
Urbanization#						
Metropolitan	5466	1542	-71.78	11.82 (11.50 to 12.13)	2.74 (2.62 to 2.86)	-6.85 (-7.70 to-5.98*)
Nonmetropolitan	1824	481	-73.62	15.98 (15.25 to 16.71)	4.55 (4.21 to 4.90)	-6.45 (-7.23 to-5.65*)
Age Group##						
55–64 years	112	82	-26.79	0.47 (0.47 to 0.47)	0.20 (0.20 to 0.20)	-4.14 (-4.72 to-3.57*)
65–74 years	491	190	-61.30	2.67 (2.67 to 2.67)	0.55 (0.55 to 0.55)	-6.47 (-6.87 to-6.07*)
75–84 years	2037	443	-78.25	16.66 (16.66 to 16.66)	2.41 (2.41 to 2.41)	-7.66 (-8.35 to-6.96*)
85 + years	4650	1307	-71.89	111.94 (111.94 to 111.94)	21.10 (21.10 to 21.10)	-6.90 (-7.44 to-6.35*)

*Statistically significant AAPC (α=0.05;P≤0.05). #For metropolitan areas, the AAMR for 2023 was unavailable and was therefore replaced with the 2020 estimate.##For age-group analyses, AAMR was calculated using age-specific (crude)mortality rates. Metro vs non-metro comparisons are based on 2020 data. Non-metropolitan estimates for 2023 are shown separately and are not directly comparable.

### Annual trend in mortality (AAMR)

Nationally, the AAMR declined from 12.67 per 100,000 in 1999 to 2.27 per 100,000 in 2023 (Table 1). This corresponded to an AAPC of − 6.87% (95% CI: −7.64 to − 6.09; *P* < 0.001), indicating a sustained decrease in mortality associated with the combined conditions over the study period (Table 1).

### Sex stratification

Among females, deaths decreased from 5,486 in 1999 to 1,384 in 2023 (− 74.77%), and the AAMR declined from 14.17 to 2.63 per 100,000 (AAPC − 6.86%, 95% CI − 7.62 to − 6.09; *P* < 0.001; Table 1). Among males, deaths decreased from 1,804 to 638 (− 64.63%), with AAMR declining from 9.14 (8.71–9.57) to 1.72 (1.59–1.86) per 100,000 (AAPC − 6.53%, 95% CI − 7.13 to − 5.92; *P* < 0.001; Table 1). Female AAMRs exceeded male AAMRs throughout the study period (1999–2023).

### Stratification by age groups

Age-specific crude death rates showed a pronounced gradient (Table 1; Fig. 1). In 1999, rates ranged from 0.47 to 111.94 per 100,000, and by 2023, rates ranged from 0.20 to 21.10 per 100,000 (Table 1). Long-term decreases were observed for all age groups (AAPC − 4.14% to − 7.66%; all *P* < 0.001; Table 1). In Joinpoint analyses (Fig. 1), aged 75–84 years showed APC − 2.08% (95% CI − 7.43 to 3.58) during 1999–2002 and APC − 8.43% (95% CI − 8.83 to − 8.03) during 2002–2023. Similarly, aged ≥ 85 years showed APC − 1.61% (95% CI − 5.91 to 2.89) during 1999–2002 and APC − 7.63% (95% CI − 7.92 to − 7.34) during 2002–2023. For younger older adults, APCs over 1999–2023 were − 4.14% (95% CI − 4.72 to − 3.57) for aged 55–64 years and − 6.47% (95% CI − 6.87 to − 6.07) for aged 65–74 years (Fig. 1).

### Ethnoracial stratification

**Fig. 2:**
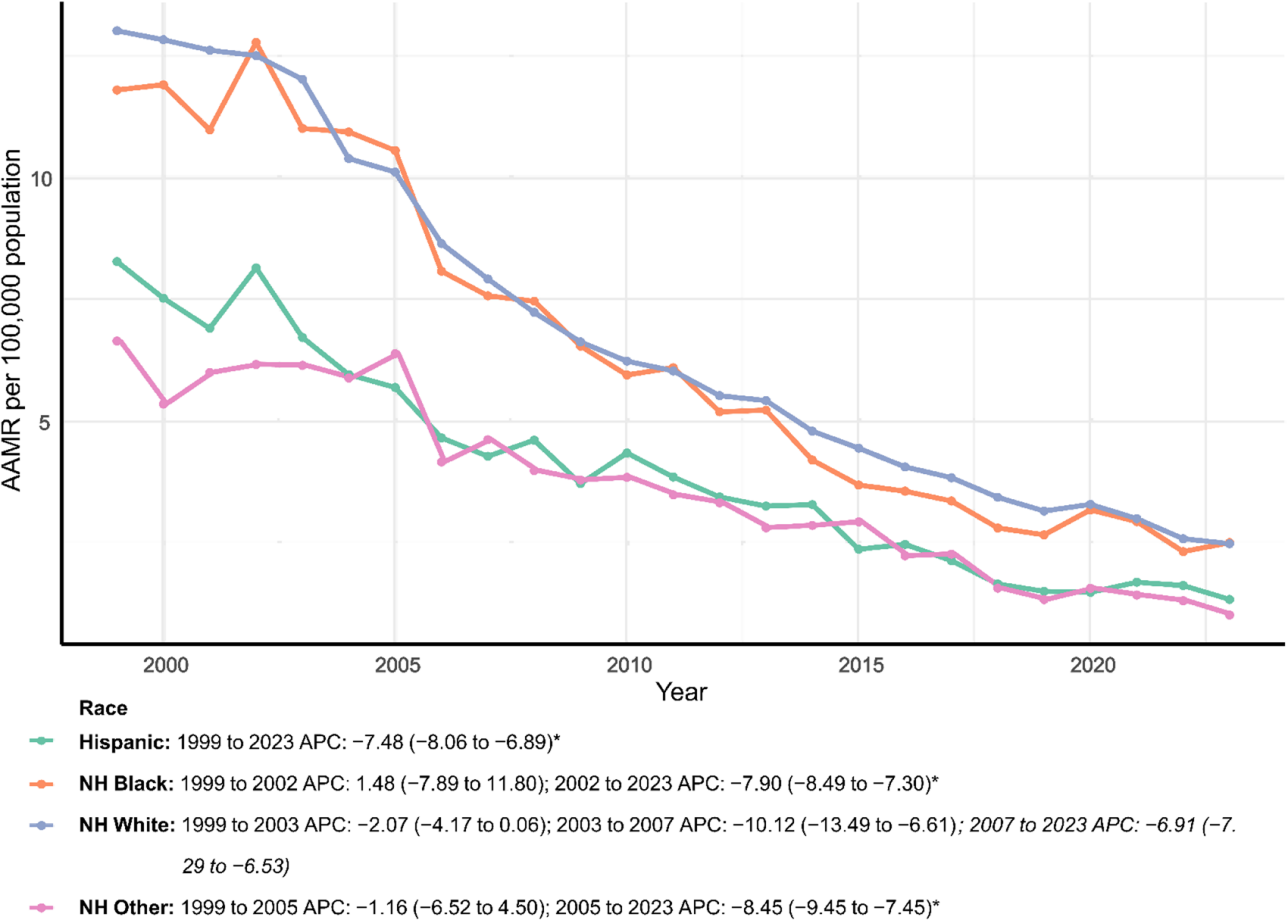
Race-stratified age-adjusted mortality rates for circulatory diseases with arthrosis, 1999–2023. Race-stratified age-adjusted mortality rates (AAMRs) per 100,000 population were calculated for deaths with an underlying cause of death of diseases of the circulatory system (ICD-10: I00–I99) and co-occurring arthrosis (ICD-10: M15–M19) among U.S.adults aged ≥ 55 years during 1999–2023. * indicates that the annual percent change (APC) is significantly different from zero atα = 0.05.AAMR, age-adjusted mortality rate; NH, non-Hispanic.

Race/ethnicity categories were harmonized across the study period using consistent definitions to ensure comparability of subgroup analyses. Across ethnoracial groups, AAMRs declined over time (all *P* < 0.001 for AAPC; Table 1; Fig. 2). NH White individuals accounted for most deaths in both 1999 (6,498) and 2023 (1,649), with AAMR decreasing from 13.01 to 2.48 per 100,000 (AAPC − 6.67%, 95% CI: −7.34 to − 5.99; Table 1). NH Black individuals had AAMR 11.80 per 100,000 in 1999 and 2.50 per 100,000 in 2023 (AAPC: −6.77%, 95% CI: −7.94 to − 5.60; Table 1). Hispanic individuals had AAMR decreasing from 8.27 to 1.34 per 100,000 (AAPC: −7.48%, 95% CI: −8.06 to − 6.89; Table 1), while NH Other individuals had AAMR decreasing from 6.64 to 1.02 per 100,000 (AAPC − 6.68%, 95% CI: −8.09 to − 5.25; Table 1). Joinpoint analyses revealed distinct changes in temporal trends across ethnoracial groups (Fig. 2): The AAMR for Hispanic individuals decreased by 7.48% per year from 1999 to 2023 (95% CI − 8.06 to − 6.89); The AAMR for NH Black individuals increased by 1.48% per year from 1999 to 2002 (95% CI − 7.89 to 11.80) and decreased by 7.90% per year from 2002 to 2023 (95% CI − 8.49 to − 7.30); The AAMR for NH White individuals decreased by 2.07% per year from 1999 to 2003 (95% CI − 4.17 to 0.06), by 10.12% per year from 2003 to 2007 (95% CI − 13.49 to − 6.61), and by 6.91% per year from 2007 to 2023 (95% CI − 7.29 to − 6.53); The AAMR for NH Other individuals decreased by 1.16% per year from 1999 to 2005 (95% CI − 6.52 to 4.50) and by 8.45% per year from 2005 to 2023 (95% CI − 9.45 to − 7.45).

### Geographic variations

**Fig. 3:**
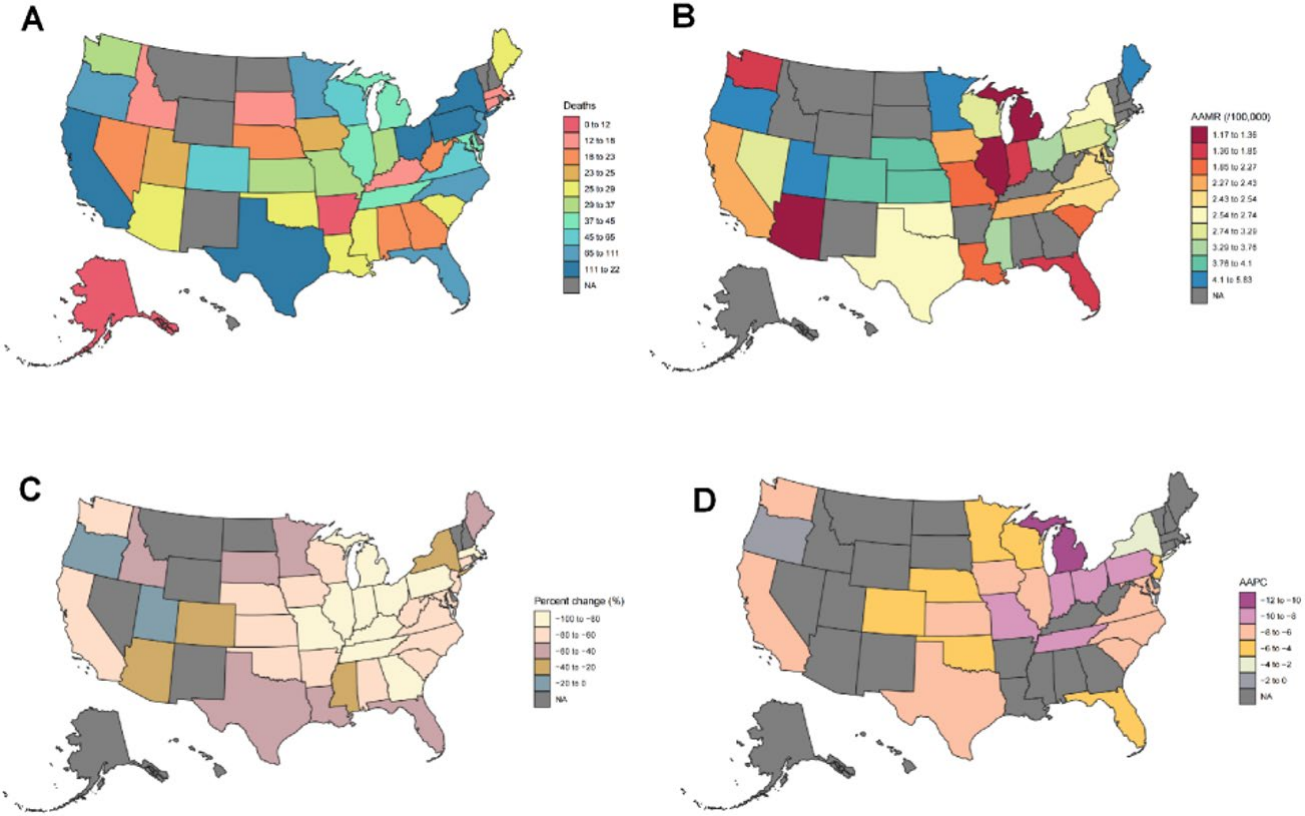
State-level variation in circulatory disease deaths with arthrosis among U.S. adults. State-level geographic variation is shown for deaths with an underlying cause of death of diseases of the circulatory system (ICD-10: I00–I99) and co-occurring arthrosis (ICD-10: M15–M19) among U.S.adults aged ≥ 55 years. (**A**) Number of deaths in 2023. (**B**) Age-adjusted mortality rates (AAMRs) per 100,000 population in 2023. (**C**) Percent change in deaths from 1999 to 2023. (**D**) Average annual percent change (AAPC) in AAMRs during 1999–2023. AAPC, average annual percent change.

State-level AAMRs showed substantial geographic variation (Fig. 3). In 1999, the highest AAMR was observed in Ohio (27.00 per 100,000), Missouri (20.61), and Pennsylvania (19.85), whereas Arizona had the lowest AAMR (3.42) (Fig. 3). By 2023, AAMRs ranged from 1.17 in Arizona to 5.83 in Oregon (Fig. 3). California contributed the largest number of deaths in both 1999 (972) and 2023 (223) (Fig. 3). State-level trends were generally decreasing; steep declines were observed in Michigan (AAPC − 10.52%, *P* = 0.000003), Indiana (AAPC − 9.15%, *P* = 0.001404), and Missouri (AAPC − 8.42%, *P* = 0.000241), whereas trends were not statistically significant in Oregon (AAPC − 1.95%, *P* = 0.270512) and Oklahoma (AAPC − 5.22%, *P* = 0.118078) (Fig. 3). Across census regions, AAMR declined from 1999 to 2023 (AAPC range: −5.92% to − 7.21%; all *P* < 0.001; Table 1). The Midwest had the highest AAMR in 1999 (17.32 per 100,000), while the Northeast had the highest AAMR in 2023 (2.54 per 100,000) (Table 1).

By urbanisation, comparisons between metropolitan and nonmetropolitan areas were restricted to 2020, the most recent year with available age-adjusted estimates for both categories. In that year, nonmetropolitan areas had higher AAMRs than metropolitan areas (Table 1). For 2023, nonmetropolitan estimates are presented separately without direct comparison because age-adjusted metropolitan estimates were not available. Per the data note, the metropolitan AAMR reported for 2023 was calculated using 2020 data (Table 1).

## Discussion

### Study summary and main findings

Using U.S. death certificate multiple-cause-of-death data, we conducted a nationwide serial cross-sectional analysis of mortality among adults aged ≥ 55 years in whom diseases of the circulatory system (I00–I99) were the underlying cause of death and arthrosis (M15–M19) was documented as a contributing condition. Multiple-cause-of-death analyses are well-suited to describing multimorbidity patterns using routinely collected vital statistics while maintaining national coverage and reproducibility^16^. Across 1999–2023, we observed a substantial decline in both deaths and AAMR, with statistically significant downward trends across most demographic and geographic strata (Table 1; Figs. 1, 2 and 3). Nevertheless, disparities persisted: mortality burden remained concentrated in the oldest age groups (Table 1; Fig. 1), females exhibited higher AAMRs than males (Table 1), nonmetropolitan areas showed consistently higher AAMRs than metropolitan areas (Table 1), and marked state-level heterogeneity was evident (Fig. 3). A key strength of this study is the use of multiple-cause-of-death data, which allows for a more comprehensive assessment of coexisting conditions and better captures the contribution of arthrosis to circulatory mortality patterns than analyses based solely on the underlying cause of death.

### Trends in the context of aging, risk factors, and prior evidence

Interpreting these findings requires acknowledging two concurrent population forces. First, cardiovascular disease continues to be a leading contributor to mortality, and national surveillance has documented long-term declines in cardiovascular mortality, albeit with periods of slowing progress^1,17^. Second, arthrosis—predominantly osteoarthritis—has expanded as a global chronic disease burden, largely driven by population aging and cardiometabolic risk factors such as obesity^3^. Within this broader context, the observed decline in circulatory-system mortality among decedents with coexisting arthrosis is broadly consistent with improvements in cardiovascular prevention and care, even as the pool of older adults living with arthrosis has grown. At the same time, the persistence of subgroup and geographic differences indicates that overall gains have not been uniform (Table 1; Figs. 1, 2 and 3).

### Subgroup patterns and plausible explanations

Subgroup findings add nuance to the overall downward trend. Sex-stratified results showed higher AAMRs among females than males (Table 1), which is compatible with the higher population burden of osteoarthritis and arthrosis in women documented in global assessments^3^. Age-stratified analyses demonstrated a steep gradient, with mortality rates remaining highest among adults aged ≥ 85 years despite large absolute reductions over time (Table 1; Fig. 1). Higher mortality among older adults is consistent with increased prevalence of multimorbidity, frailty, and cumulative cardiovascular risk. Race/ethnicity-stratified trends indicated persistent differences in both levels and slopes (Table 1; Fig. 2). Differences across racial/ethnic groups may reflect structural, socioeconomic, and healthcare access disparities in the U.S. These disparities may reflect differences in healthcare access, availability of preventive cardiovascular services, socioeconomic conditions, and the distribution of cardiometabolic risk factors across populations. Geographic variation was pronounced: nonmetropolitan areas retained higher AAMRs than metropolitan areas in both earlier and later periods (Table 1), and state-level mapping highlighted substantial heterogeneity in contemporary burden (Fig. 3). These patterns plausibly reflect regional differences in cardiovascular risk factor prevalence, access to preventive and acute cardiovascular care, and broader contextual determinants (e.g., socioeconomic conditions and healthcare infrastructure). Because our data are ecological and based on death certificate coding, these potential explanations should be interpreted as hypotheses rather than causal inferences.

### Implications, NSAID context, and limitations

From a clinical and public health perspective, our findings support integrated approaches to cardiovascular risk assessment and management among older adults with arthrosis, particularly in settings and subgroups with persistently higher mortality burden (Table 1; Fig. 3). In addition, the intersection of arthrosis care and cardiovascular risk is clinically salient. Although NSAID use is clinically relevant in the management of arthrosis, our analysis did not include individual-level medication data and cannot assess its association with mortality. Future studies integrating clinical or claims data may help clarify this relationship. Several limitations warrant consideration. Death certificate data are subject to misclassification, and arthrosis may be under-recorded as a contributing condition, which could lead to underestimation of co-occurrence. Multiple-cause-of-death analyses reflect co-mention on the death certificate rather than clinical temporality; therefore, they cannot establish directionality or causal pathways linking arthrosis and circulatory-system mortality^16^. Because our analysis is based on U.S. death certificate data, we were unable to adjust for individual-level clinical characteristics, comorbidities, health care access, or medication use, and residual confounding is possible. These limitations reflect the inherent constraints of death certificate data rather than CDC WONDER specifically. While race/ethnicity classifications changed over time, urbanization classifications were applied consistently using the 2006 or 2013 NCHS schemes, minimizing the impact on comparability across years. In addition, CDC WONDER did not return an age-adjusted metropolitan estimate for 2023 at the time of extraction; the metropolitan value shown for the most recent year corresponds to the most recent available estimate (2020), as noted in Table 1. Despite these constraints, the national scope and long time series provide a robust epidemiologic description of evolving circulatory-system mortality patterns in the presence of arthrosis among older U.S. adults.

## Conclusion

Using CDC WONDER multiple-cause-of-death data (1999–2023), we quantified U.S. mortality among adults aged ≥ 55 years with diseases of the circulatory system (I00–I99) as the underlying cause of death and arthrosis (M15-M19) documented as a coexisting condition. Mortality burden declined substantially over the study period, yet meaningful heterogeneity persisted across age, sex, race/ethnicity, and geography, including higher rates in nonmetropolitan settings and marked state-level variation. These findings support continued surveillance and may inform targeted cardiovascular risk assessment and management strategies in older adults with arthrosis.

## Data Availability

The data analysed in this study are publicly available from the Centres for Disease Control and Prevention (CDC)Wide-ranging Online Data for Epidemiologic Research (CDC WONDER) Multiple Cause of Death database. Aggregated mortality data can be accessed and downloaded through the CDC WONDER web interface. No individual-level, identifiable data were obtained or generated for this study.
